# PD23 Economic Evaluation Of Prophylactic Immunoglobulin Versus Prophylactic Antibiotics In Hematological Malignancies: Results From The RATIONAL Feasibility Trial

**DOI:** 10.1017/S0266462324002873

**Published:** 2025-01-07

**Authors:** Sara Carrillo De Albornoz, Alisa Higgins, Dennis Petrie, Adam Irving, Laura Fanning, Robert Weinkove, Philip Crispin, Claire Dendle, Michael Gilbertson, Anna Johnston, Anastazia Keegan, Dominic Pepperell, Humphrey Pullon, John Reynolds, Tina van Tonder, Judith Trotman, Neil Waters, Cameron Wellard, Helen Weston, Orla Morrissey, Erica Wood, Zoe McQuilten

## Abstract

**Introduction:**

Patients with hematological malignancies are at high risk of infections due to both the disease and the associated treatments. The use of immunoglobulin (Ig) to prevent infections is increasing in this population, but its cost effectiveness is unknown. This trial-based economic evaluation aimed to compare the cost effectiveness of prophylactic Ig with prophylactic antibiotics in patients with hematological malignancies.

**Methods:**

The economic evaluation used individual patient data from the RATIONAL feasibility trial, which randomly assigned 63 adults with chronic lymphocytic leukemia, multiple myeloma, or lymphoma to prophylactic Ig or prophylactic antibiotics. The following two analyses were conducted to estimate the cost effectiveness of the two treatments over the 12-month trial period from the perspective of the Australian health system:

(i) a cost-utility analysis (CUA) to assess the incremental cost per quality-adjusted life-year (QALY) gained using data collected with the EuroQol 5D-5L questionnaire; and

(ii) a cost-effectiveness analysis (CEA) to assess the incremental cost per serious infection prevented (grade ≥3) and per infection prevented (any grade).

**Results:**

The total cost per patient was significantly higher in the Ig arm than in the antibiotic arm (difference AUD29,140 [USD19,000]). There were non-significant differences in health outcomes between the treatment arms: patients treated with Ig had fewer QALYs (difference −0.072) and serious infections (difference −0.26) than those given antibiotics, but more overall infections (difference 0.76). The incremental cost-effectiveness from the CUA indicated that Ig was more costly than antibiotics and associated with fewer QALYs. In the CEA, Ig costed an additional AUD111,262 (USD73,000) per serious infection prevented, but it was more costly than antibiotics and associated with more infections when all infections were included.

**Conclusions:**

These results indicate that, on average, Ig prophylactic treatment may not be cost effective compared with prophylactic antibiotics for the group of patients with hematological malignancies recruited to the RATIONAL feasibility trial. Further research is needed to confirm these findings in a larger population and over the longer term.

